# Cracking the Codes for Congenital Diarrhea and Enteropathies (CoDEs): A Case Report and Review

**DOI:** 10.7759/cureus.73529

**Published:** 2024-11-12

**Authors:** Anushka Bhowal, Natalia Cardona, Caroline Chua, Sreekanth Viswanathan, Jolanda Denham, Lili Miles, James P Franciosi

**Affiliations:** 1 Pediatrics, Nemours Children's Health, Orlando, USA; 2 Neonatology, Nemours Children's Health, Orlando, USA; 3 Gastroenterology, Hepatology and Nutrition, Nemours Children's Health, Orlando, USA; 4 Pathology, Nemours Children's Health, Orlando, USA; 5 Pediatrics, University of Central Florida College of Medicine, Orlando, USA

**Keywords:** congenital diarrhea and enteropathy, dehydration, failure to thrive, glucose galactose malabsorption, neonate

## Abstract

Congenital diarrhea and enteropathies (CoDEs) condition is a rare cause of chronic diarrhea in infants that can be challenging to diagnose. This article discusses key signs to recognize in considering a CoDEs diagnosis and provides an overview of the diagnostic process.

We report a late preterm twin infant with intractable watery diarrhea starting shortly after birth. The infant was born from a twin pregnancy to non-consanguineous parents with an unremarkable family history. His twin brother had no complications after birth and continued to thrive. The patient initially presented with bloody stools, leading to a suspected diagnosis of necrotizing enterocolitis or cow’s milk protein-induced allergic colitis. However, the emergence of profuse and watery diarrhea, failure to thrive, and hypernatremic dehydration shifted the suspicion toward malabsorptive diarrhea.

An extensive workup was significant for hypernatremic metabolic acidosis and positive stool-reducing substances. Several trials of protein-hydrolysate and elemental amino acid-based formulas failed to improve symptoms. However, stool consistency improved with a trial of a carbohydrate-free, hydrolyzed protein-based formula (3232A). As a diagnostic test for specific carbohydrate malabsorption, the infant was challenged with glucose supplementation followed by fructose supplementation; his stool consistency worsened with the glucose challenge but improved with the fructose challenge. His stool pH and reducing substances were abnormal after the glucose challenge and normalized after the fructose challenge, thus indicating a clinical diagnosis of glucose-galactose malabsorption (GGM).

At the time of discharge, the infant had documented weight gain and formed stools on carbohydrate-free, hydrolyzed protein-based formula (3232A) supplemented with fructose. At one-year follow-up after discharge, he continued to thrive with normal bowel movements. Outpatient genetic testing confirmed our diagnosis of GGM.

GGM should be considered in infants with severe protracted, non-infectious, watery diarrhea lasting longer than two weeks. Early diagnosis and management of infants with GGM with a carbohydrate-free formula with specific carbohydrate supplementation are essential to prevent complications and ensure optimal growth and development.

## Introduction

Congenital diarrhea and enteropathies (CoDEs) condition is a rare cause of chronic diarrhea in infants that can be challenging to diagnose. Glucose galactose malabsorption (GGM) is a genetic CoDEs disorder caused by a mutation in the solute carrier family five member-1 gene and resulting in the nonfunction of the sodium-dependent glucose transporter-1 [1]. Disruption of glucose and galactose transport across the intestine brush border membrane results in the accumulation of unabsorbed galactose and glucose in the intestinal lumen. Neonates present with significant osmotic diarrhea and dehydration soon after birth. There are an estimated 300 reported cases worldwide. This case underscores the complexity of making a diagnosis in this spectrum of congenital disorders, particularly in the presence of non-specific symptoms such as hypoglycemia, failure to thrive, and persistent diarrhea, which overlap with more common neonatal conditions. Through genetic analysis and a stratified diagnostic approach, this report highlights the challenges and strategies in identifying this rare group of genetic disorders in the neonatal period.

## Case presentation

This is a male infant of diamniotic/dichorionic twin gestation (twin B) born via spontaneous vaginal delivery to a 37-year-old G5, P 1 full-term, two pre-term (twins), three abortions, three live births mother at 36 weeks and two days. The mother had an unremarkable pregnancy with normal prenatal screenings and negative serologies. There was no known family history of genetic disorders or chronic gastrointestinal disease. At delivery, the neonate was vigorous; however, he soon developed signs of mild respiratory distress syndrome, necessitating admission to the neonatal intensive care unit. The infant briefly required noninvasive positive pressure ventilation but was able to be weaned to unassisted room air by the third day of life. He received 48 hours of ampicillin and gentamicin to rule out sepsis/pneumonia as a standard of care. During the remainder of the infant's admission, notable issues included the production of loose stools and ongoing hypoglycemia despite continuous fortified feeds. His diarrhea and hypoglycemic episodes were both resolved by the time he was discharged on the 17th day of life. The patient was taking fortified breast milk of 22 kcal/oz with standard preterm formula at the time of discharge.

**Figure 1:**
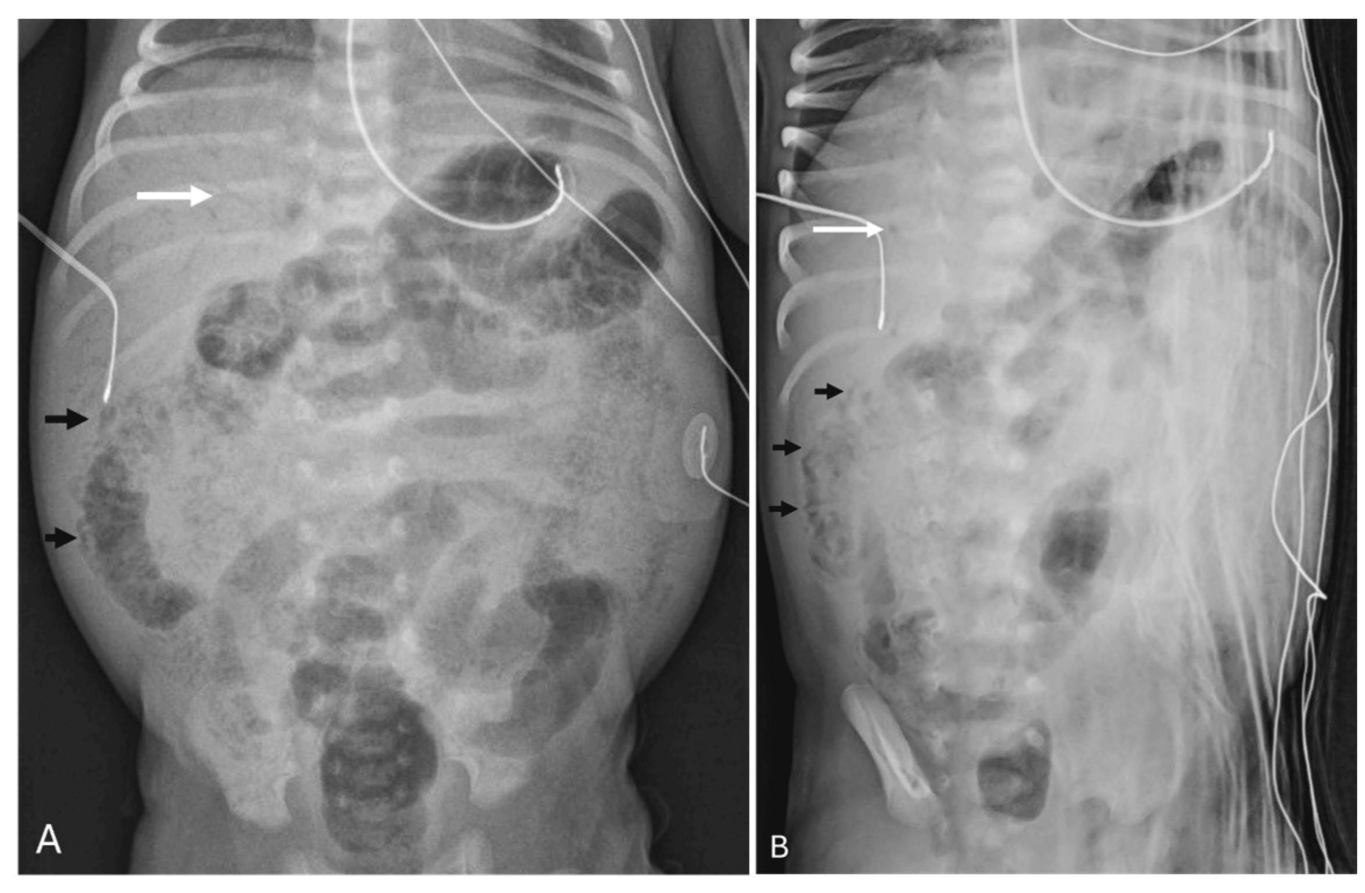
Diffuse pneumatosis and portal venous gas Panels A and B depict diffuse pneumatosis and portal venous gas.

Three days later, the patient presented to the emergency department due to inconsolability and frank bloody stools. His breast milk fortification was increased from 22 kcal/oz to 24 kcal/oz the day before presentation. Laboratory evaluation included a normal complete blood count and lactate level. An abdominal radiograph was concerning for signs of necrotizing enterocolitis (NEC) with diffuse pneumatosis and portal venous gas (Figure 1).

On exam, the infant was lethargic with a sunken fontanelle, tachycardic, and tachypneic, with marked abdominal distention and hypoactive bowel sounds. Broad antimicrobial coverage with piperacillin/tazobactam and vancomycin was employed on admission; however, all infectious work-ups, including complete blood count, C-reactive protein, blood, and urine cultures, returned negative. The patient was kept nil per os and received total parenteral nutrition for two days, during which time his bloody stools resolved. Serial abdominal imaging showed complete resolution of pneumatosis and portal venous gas. Oral feeds with an elemental amino acid-based formula were introduced with excellent response, so the patient was empirically diagnosed with cow’s milk protein-induced allergic colitis at discharge.

Five days later, the patient presented again to the emergency department when his mother noted blood clots in his stools. In the week after discharge and prior to presentation, the patient had watery stools with incremental weight loss despite the infant feeding vigorously. The initial work-up with complete blood count, complete metabolic panel, and gastrointestinal polymerase chain reaction panel were negative. As part of the standard diagnostic workup, a viral respiratory polymerase chain reaction was performed. The infant tested positive for parainfluenza, but this was felt to be an incidental finding as the infant did not have any respiratory symptoms. An abdominal radiograph once again showed signs of pneumatosis concerning for NEC; the stool occult blood was positive. Upon admission, the patient remained nil per os on total parental nutrition for the first 12 hours and was transitioned to an amino acid-based formula, during which his bloody stools completely resolved. However, he continued to have profuse watery diarrhea with concomitant weight loss despite the infant feeding up to 200 mL/kg/day. His total fluid goal was decreased to 170 mL/kg/day to reduce stool output; however, the patient continued to have 10 to 11 large-volume, dilute stools per day. Pectin was added to the patient’s formula and treatment was initiated with metronidazole for small bowel bacterial overgrowth, neither of which made any difference in stool output or consistency. The patient ultimately developed hypernatremic dehydration with a maximum sodium level of 168 mmol/L and a blood urea nitrogen level of 54 mg/dL, necessitating a return to parenteral nutrition.

**Figure 2:**
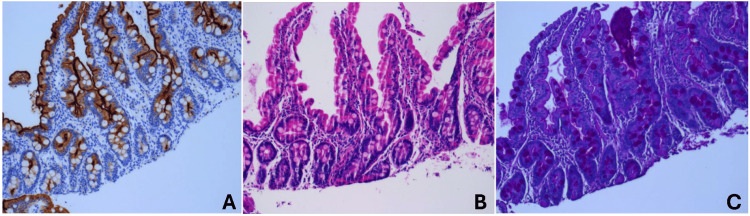
Light microscopic examination of the duodenum Light microscopic examination of the duodenum revealed preserved villous and cryptal architecture without active cryptitis or increase of intraepithelial lymphocytes; panel A  CD10 staining, panel B  hematoxylin and eosin, and panel C Periodic acid-Schiff staining.

A work-up was initiated for suspected malabsorptive diarrhea, given that the patient’s stool output and consistency worsened with enteral feeds but improved during nil per os periods. The patient underwent an upper gastrointestinal series with small bowel follow-through, an endoscopy with flexible sigmoidoscopy, and a barium enema, all of which were normal. Biopsies were sent for pathology review and disaccharidase analysis, which were both normal (Figure 2). Stool alpha-1-antitrypsin level and fecal elastase were normal. Stool studies with stool electrolytes and osmolality were also normal. However, there was the presence of reducing substances in the stool and low stool pH raising the concern for carbohydrate malabsorption.

A feeding trial with carbohydrate-free hydrolyzed protein-based formula (3232A®, Mead Johnson, Chicago, IL) was initiated, and the patient experienced marked improvement in stool output and consistency within 24 hours of initiation. Suspecting a diagnosis of GGM, glucose, and fructose challenges were conducted whereby the patient was fed carbohydrate-free formula first with glucose supplementation mixed into feeds and then a separate trial with fructose supplementation. Stool pH and reducing substances were collected after each challenge. After the glucose challenge, the stool pH was 5.0 with the presence of stool-reducing substances. After the fructose challenge, the stool pH was 6.5 with negative stool-reducing substances. The patient was discharged with a clinical diagnosis of GGM on carbohydrate-free formula plus fructose to maintain normoglycemia, on which he continues to thrive. A congenital diarrhea genetic panel returned several months later, with the patient identified to have two missense variants, SLC5A1 c.1028T>C, p.(Ile343Thr) and SLC5A1 c.1063T>A, p.(Cys355Ser). Because both variants are defined as loss of function with only one allele needing to be pathogenic (compound heterozygous), a genetic diagnosis of GGM supporting the clinical presentation was confirmed.

At one year of age, the patient continues to be on carbohydrate-free formula supplemented with fructose but has been able to incorporate solids into his diet under the guidance of a registered dietician. He continues to have adequate weight gain and linear growth with a consistent trend at the 52nd percentile for weight-for-length.

## Discussion

Chronic diarrhea is defined as diarrhea that is persistent for more than two weeks. CoDEs should be considered as part of the differential when all other secondary causes, including allergic, anatomical, and infectious causes have been ruled out [2]. GGM is an autosomal recessive disorder caused by a mutation in the SLC5A1 gene on chromosome 22q13.1, which leads to a loss of function of the sodium/glucose cotransporter (SGLT1) [3,4]. Most cases occur in ethnic groups with consanguineous parents or in geographic areas with founder effects [5]. There are currently 63 mutations in SLC5A1 leading to congenital GGM listed in the Human Gene Mutation Database. Our patient has the Cys355Ser mutation, which has been shown to eliminate sodium/glucose cotransport by blocking the transfer of SGLT1 protein from the endoplasmic reticulum to the plasma membrane [4]. Early signs of CoDEs can be mistaken for acquired diarrhea due to other presenting symptoms. GGM should be considered in infants with severe, protracted, non-infectious watery diarrhea lasting longer than two weeks [2]. Early diagnosis and management of infants with GGM with a carbohydrate-free formula with fructose supplementation are essential to prevent complications and ensure optimal growth and development.

**Figure 3:**
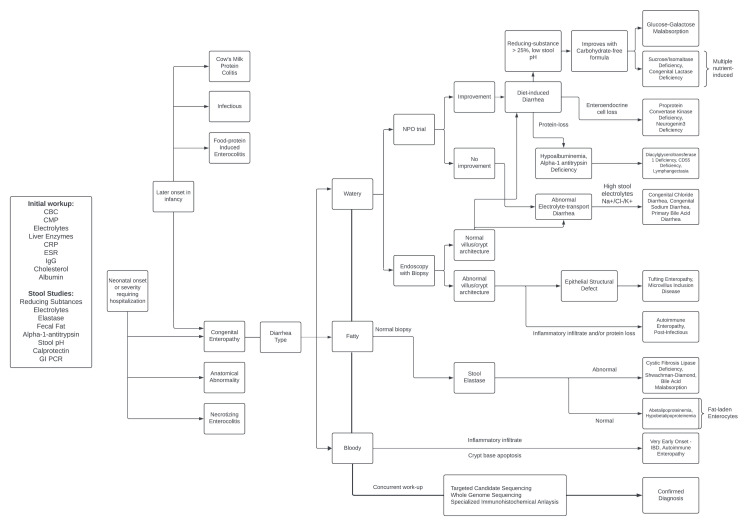
CoDEs diagnostic algorithm Reprinted and modified from [2] with permission from Elsevier. CBC, complete blood count; CMP, comprehensive metabolic panel; CoDEs, congenital diarrhea and enteropathies; CRP, C-reactive protein; ESR, erythrocyte sedimentation rate; GI, gastrointestinal; IBD, inflammatory bowel disease; IgG, immunoglobulin G; PCR, polymerase chain reaction; NPO, nil per os.

An algorithm for differential diagnosis and workup for diarrhea in the neonatal and early infancy period based on the algorithm created by Thiagarajah et al. can be found in Figure 3 [2].

For our patient, after all secondary causes were addressed, the patient persisted with profuse watery stools, hypoglycemic episodes, and inconsistent weight gain. Only after multiple admissions and attempts with different formulas were unsuccessful, a CoDEs diagnosis was suspected, and subsequent studies were sent.

Although genetic studies are important to obtain, clinical investigation with fasting trials and observation of symptoms with intake of carbohydrate-free formula should occur concurrently to obtain a clinical diagnosis [1,2,6]. In our patient, his feeding intolerance, weight loss, and stool consistency improved with a trial of a carbohydrate-free, hydrolyzed protein-based formula (3232A®). This formula is used in the dietary management of infants and children with disaccharidase deficiencies or other disorders of carbohydrate metabolism. The 3232A® formula contains tapioca starch and is considered an incomplete source of carbohydrates and must be used with added carbohydrates (e.g., glucose, fructose). 

As a clinical diagnostic test for specific carbohydrate malabsorption, the infant was then challenged with specific carbohydrates of glucose supplementation followed by fructose supplementation while being maintained on 3232A® formula; his stool consistency worsened with the glucose challenge but improved with the fructose challenge. His stool pH and reducing substances were abnormal after the glucose challenge and normalized after the fructose challenge, thus indicating a clinical diagnosis of GGM.

In this patient, the initial presentation of hypoglycemia and loose stools in the first few days of life, interjected by isolated episodes of frank bloody stools prior to the true development of intractable, voluminous watery diarrhea, confounded and delayed formal investigation into a CoDEs diagnosis [7]. 

On review, other cases of GGM in the literature classically present with persistent watery diarrhea, failure to thrive, and early feeding intolerance that were not initially present in this patient [1]. His presentation was confounded with frank bloody diarrhea, pneumatosis intestinalis, and portal venous gas. We suspected cow's milk allergy because patients with NEC presenting with portal venous gas and pneumatosis are significantly sicker with systemic symptoms. 

We believe that in this case, the patient’s resolution of symptoms during periods of bowel rest, in addition to clinical improvement with intake of hypoallergenic and amino acid formula, resulted in a low index of suspicion for congenital enteropathies. Furthermore, multiple admissions for a premature infant presenting with frank bloody stool and lethargy responding to bowel rest and antibiotics resulted in an anchoring bias for the diagnosis of NEC [8]. In our case, compared with classic NEC presentations, the infant was clinically less sick, and in both occasions of bloody stools, enteral feeds were introduced within 24-48 hours with no further gastrointestinal concerns for NEC. NEC is also more common in more premature infants. Considering this infant was born at 36 weeks, we considered cow's milk protein-induced allergic colitis as the first diagnosis. The cow's milk protein allergy was an empirical diagnosis as no specific test was performed. The diagnosis of cow's milk protein-induced allergic colitis is often based on clinical response to a milk elimination diet. Although uncommon, there are previous reports of cow's milk allergy presenting with portal venous gas/pneumatosis [9,10]. Interestingly, co-existing cow's milk protein-induced allergic colitis with GGM was suspected with the return of bloody stools with a challenge of a soy-based carbohydrate formula (Ross Carbohydrate Free®, Abbot Nutrition, Chicago, IL), and then clinically confirmed with tolerance of a carbohydrate-free, hydrolyzed protein-based formula (3232A®).

Taken together, the gold standard approach for diagnosing CoDEs includes whole exome sequencing and specific molecular analysis [1,2,6]. Genetic testing revealed that our patient is compound heterozygous with two missense mutation variants of the SLC5A1 gene. Our patient has the SLC5A1 c.1063T>A, p.(Cys355Ser) variant, which has been shown to eliminate the sodium/glucose cotransport by blocking the transfer of SGLT1 protein from the endoplasmic reticulum to the plasma membrane [4]. Interestingly, SGLT1 has also been shown to have an immunomodulator role. Activation of the SGLT1 protein results in inhibition of IL-8-derived chemokine production and inactivation of Toll-like receptors and subsequent NF-kB transcription [11]. Our patient has a non-functioning SGLT1 protein, which may point to an upregulated proinflammatory process resulting in multiple episodes resembling NEC.

Limitations of this case report include a small pool of patients with similar clinical presentations. Approximately 300 cases have been reported in the literature, and mostly in ethnic groups with consanguinity present [3]. From our review, little to no data are reported on cases as a product of non-consanguineous pregnancy or twin pregnancies. There is only one other case describing a patient in Brazil with GGM who presented similarly with recurring NEC and was born to presumed non-consanguineous parents [12]. To our knowledge, our case is unique since our patient is the product of a non-consanguineous twin pregnancy with parents of differing racial backgrounds.

## Conclusions

In conclusion, patients with persistent watery diarrhea should be tested for acquired causes in addition to congenital causes of diarrhea when symptoms seem inconsistent. After excluding infectious, anatomic, and allergic causes of diarrhea, investigation for genetic and congenital diarrhea and enteropathies should be considered. Evaluation of enteropathies should be guided by stool consistency (watery, fatty, bloody) to help narrow down the differential with the caveat that overlap can occur. Histopathological and molecular analysis may be required for diagnosis. An early fasting trial and early gene testing are crucial in the diagnosis of CoDEs. The absence of genetic disorders or consanguinity in family history should not be a deterrent to investigating a CoDEs diagnosis. Treatment for GGM is the removal of glucose and galactose from the diet with fructose supplementation to ensure optimal growth and development.

## References

[1] Younis M, Rastogi R, Chugh A, Rastogi S, Aly H (2020). Congenital diarrheal diseases. Clin Perinatol.

[2] Thiagarajah JR, Kamin DS, Acra S (2018). Advances in evaluation of chronic diarrhea in infants. Gastroenterology.

[3] Babcock SJ, Flores-Marin D, Thiagarajah JR (2023). The genetics of monogenic intestinal epithelial disorders. Hum Genet.

[4] Martín MG, Lostao MP, Turk E, Lam J, Kreman M, Wright EM (1997). Compound missense mutations in the sodium/D-glucose cotransporter result in trafficking defects. Gastroenterology.

[5] Pomerance HH (1997). Nelson textbook of pediatrics. Arch Pediatr Adolesc Med.

[6] Kijmassuwan T, Balouch F (2024). Approach to congenital diarrhea and enteropathies (CODEs). Indian J Pediatr.

[7] Nowak-Węgrzyn A, Katz Y, Mehr SS, Koletzko S (2015). Non-IgE-mediated gastrointestinal food allergy. J Allergy Clin Immunol.

[8] Cordova J, Sriram S, Patton T, Jericho H, Gokhale R, Weinstein D, Sentongo T (2016). Manifestations of cow's-milk protein intolerance in preterm infants. J Pediatr Gastroenterol Nutr.

[9] Siddique Z, Thibodeau R, Jafroodifar A, Hanumaiah R (2021). Pediatric milk protein allergy causing hepatic portal venous gas: case report. Radiol Case Rep.

[10] Carvalho AA, Faustino J, Bota S, Ferreira ST (2021). Unusual presentation in cow's milk protein allergy. BMJ Case Rep.

[11] Palazzo M, Gariboldi S, Zanobbio L (2008). Sodium-dependent glucose transporter-1 as a novel immunological player in the intestinal mucosa. J Immunol.

[12] Mergener R, Nunes MR, Nascimento LPC, Muniz VF, Graziadio C, Zen PRG (2024). Congenital glucose-galactose malabsorption: a case report about cause and consequence, not exactly in this order. Global Pediatrics.

